# Current Strengths and Limitations of Toxicokinetic-Toxicodynamic (TKTD) Models for Freshwater Microalgae

**DOI:** 10.1021/acs.est.6c05753

**Published:** 2026-10-07

**Authors:** Emma Yenney, Nina Cedergreen

**Affiliations:** Department of Plant and Environmental Sciences, University of Copenhagen, Thorvaldsensvej 40, 1871 Frederiksberg C, Denmark

**Keywords:** Toxicokinetic toxicodynamic modeling, freshwater microalgae, environmental risk assessment, time variable exposure, population modeling

## Abstract

Toxicokinetic-toxicodynamic (TKTD) models are increasingly used to enhance environmental risk assessment by linking laboratory toxicity data with dynamic exposure scenarios. For freshwater microalgae, which are crucial primary producers, TKTD approaches offer a framework to predict population growth inhibition and recovery under fluctuating chemical stress. A review of 12 studies (2012–2023) applying TKTD models to freshwater planktonic microalgae was conducted to compare their structural assumptions, end points, and validation methods. These models mainly focus on green algae and herbicides that inhibit photosystem II, with limited exploration of other taxonomic groups or modes of action. The complexity of models varies significantly, from simple structures assuming rapid equilibrium between external and internal chemical concentrations to mechanistic models that explicitly incorporate TK components and damage progression. While simple models support regulatory use, omitting TK processes can reduce their effectiveness for chemicals with delayed effects or complex uptake behaviors. Expanding taxonomic coverage and enhancing external validation under realistic exposure conditions is crucial to increasing the ecological relevance of algal TKTD modeling.

## Graphical abstract

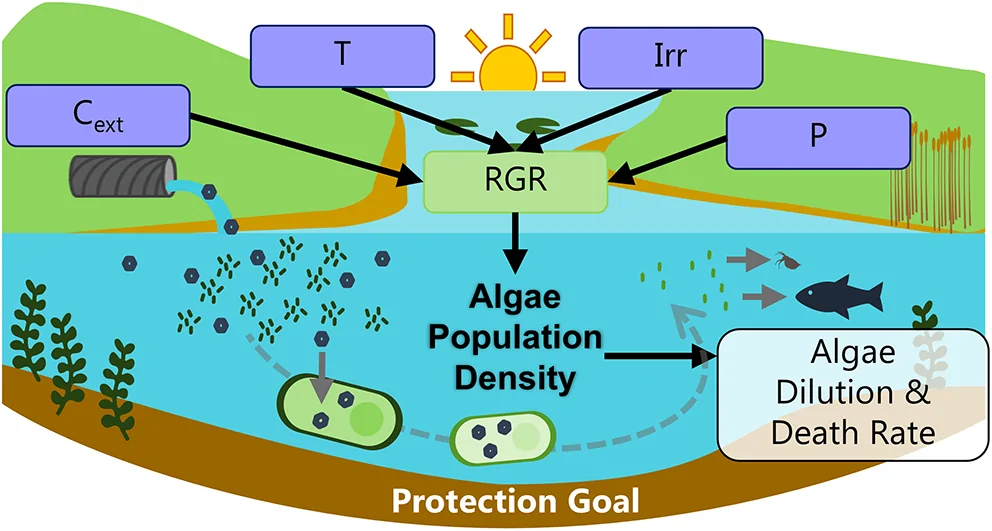

## Introduction

As new pesticides, pharmaceuticals, and industrial chemicals continue to enter the market, the need for thorough environmental risk assessment (ERA) is more prevalent than ever. In the European Union (EU), ERA is conducted under different regulatory frameworks, including (EC) No 1107/2009 ^1^ for plant protection products. Across these frameworks, ERA generally involves an exposure assessment and an effect assessment, which are compared using conservative safety factors to ensure environmental protection.^2^ Standardized laboratory toxicity tests form the basis of effect assessment and typically measure concentration response relationships under constant exposure conditions and at a fixed exposure time. However, real world exposure patterns are often variable, characterized by short pulses of high concentration followed by recovery periods, especially for pesticides. These temporal dynamics are not fully represented in standard Tier 1 risk assessment laboratory tests, introducing uncertainty into risk predictions.^2^

Toxicokinetic-toxicodynamic (TKTD) modeling has become a promising approach to connect laboratory exposure and fluctuating environmental conditions to make more realistic predictions.^1,2^ TKTD models combine toxicokinetics (uptake, elimination, biotransformation) with toxicodynamics (damage, repair, and resulting biological effects) to predict organism responses under dynamic exposure conditions. For pesticides where exposures are driven by application patterns and rainfall events, incorporating temporal dynamics may improve realism compared to static concentration response approaches. Freshwater microalgae are key primary producers and are often among the most sensitive taxa to herbicides and other chemicals.^3,4^ Reductions in algal biomass can cascade through food webs,^5^ affect oxygen production,^6^ and alter ecosystem biodiversity.^7^ Since many phytoplankton species are unicellular and exhibit rapid population turnover,^8^ they are especially responsive to short-term exposure pulses and recovery periods. These characteristics separate algae chemical effects models from other effect models, such as the primary producer *Lemna sp*. TKTD model,^9^ or survival models such as the General Unified Threshold model for Survival (GUTS)^10^ or energy fluxes in models such as Dynamic Energy Budget (DEB).^11^ Figure 1 conceptualizes TKTD modeling for freshwater microalgae, linking external exposure, internal kinetics, cellular damage, and population level growth under time variable conditions.

**Figure 1:**
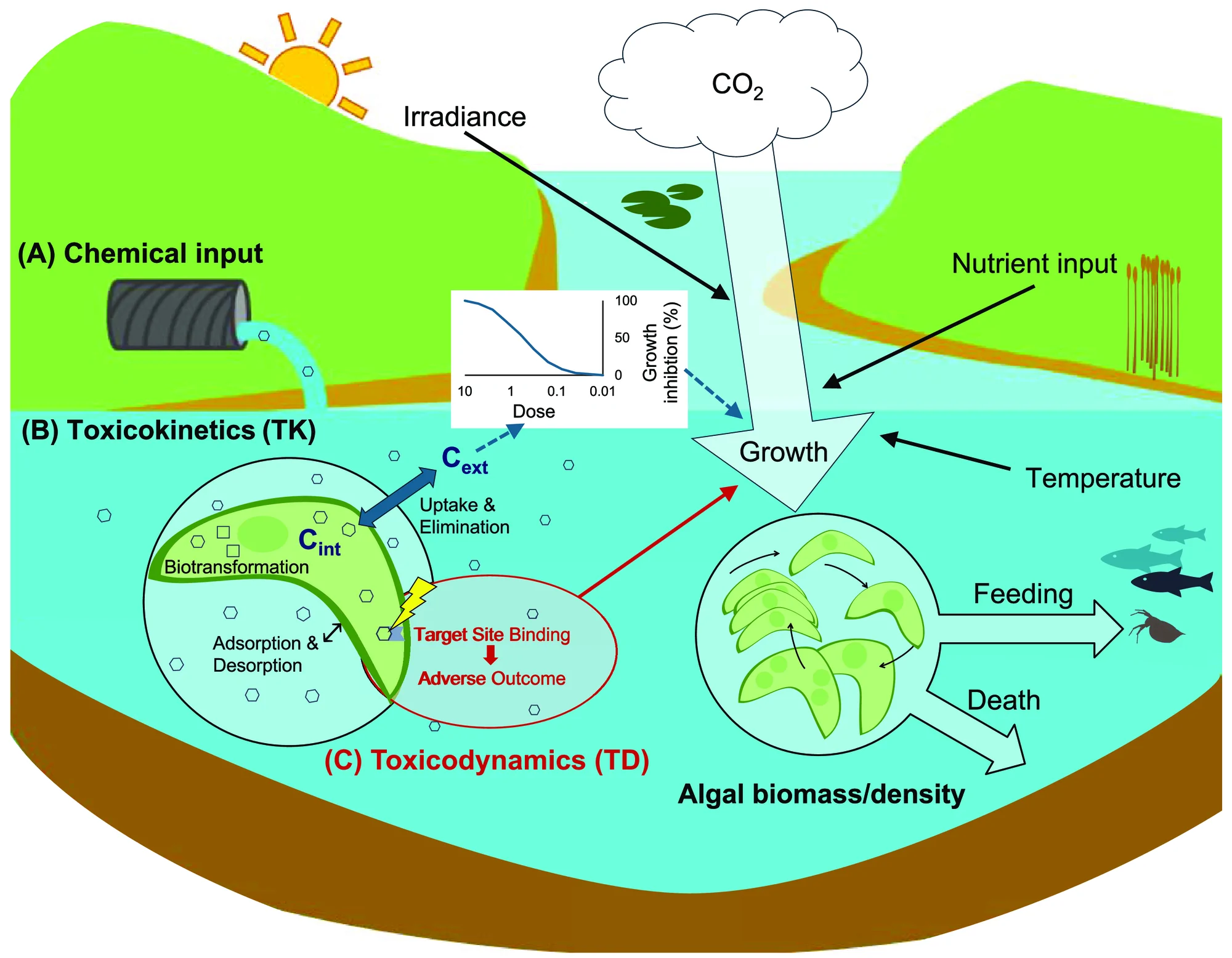
Conceptual overview of toxicokinetic-toxicodynamic (TKTD) modeling for freshwater microalgae in a natural environment. Any TKTD model will always consist of (at least) two lines of material flow: The first is the growth model where algae fix CO_2_ into algae biomass, which is again lost due to natural background mortality and to other loss rates as e.g., animals feeding on the algae (death/feeding arrows). The growth model will also depend on other factors/forcings such as irradiance, temperature and nutrient inputs, given in black arrows. The other material flow is the chemical being released from its source (A). From the water, the chemical is taken up by the algae, being biotransformed and/or excreted (Blue filled arrow). Jointly these are the toxicokinetic processes (B). The internal concentrations will elicit damage by binding to biological targets, starting a cascade of adverse effects jointly summed into Adverse Outcome Pathways (AOP), which can be described at different levels of detail (C). These toxicodynamic effects can interfere with key cellular functions, such as photosynthesis, protein integrity, DNA replication, mitotic progression, and cytokinesis, depending on the chemical mode of action. The disruptions are incorporated into TD models to describe reductions in growth rates and are collectively given in red writing, with a red arrow signifying effects on growth. Some models skip the TKTD modules entirely and relate external concentrations to decrease in growth rates directly (Broken line arrow). We call them “direct effects models” and this is what we are used to seeing as concentration–response curves using a concentration at a given time point.

The added value of TKTD models is their ability to simulate fluctuating exposure scenarios, the resultant delayed or irreversible effects, and recovery dynamics.^12^ Mechanistic models can help explain observed algae growth inhibition from internal concentration dynamics,^13^ effect progression,^14^ or cell cycle processes.^15^ This can improve scientific understanding and, in some cases, reduce reliance on conservative assessment factors by providing refined predictions under realistic exposure. However, TKTD models require additional parameters compared to traditional concentration–response curves, such as time-resolved experimental data, and data sets for model validation.^16^ Increased mechanistic complexity may introduce parameter uncertainty and reduce transparency if not carefully implemented. Currently, not all regulatory questions require mechanistic depth. For Tier 1–2 risk assessment, simpler growth rate based models may be sufficient and more practical.^16^

For regulatory Tier 2C applications, the simplified algae population model extended, SAM-X, has been proposed to use data from standard OECD growth inhibition tests^17^ to calibrate models that can predict the effect of time variable exposure scenarios, while maintaining static laboratory conditions as inputs.^2,16,16^ In this context, the objective is not to model the full complexity of the ecosystem but to refine exposure effect relationships transparently for functional protection. Alternatively, more mechanistic TKTD models help identify which biological processes drive observed toxicity patterns and under which chemical modes of action (MoA), assumptions of instantaneous concentration equilibrium may fail.

This review evaluates the current state of TKTD modeling for freshwater microalgae with four objectives: summarize the existing literature and modeling approaches, identify which biological and chemical parameters have been successfully incorporated, provide guidance for model selection, calibration, and validation, and highlight the limitations and gaps in the current models for future research. These four objectives aim to improve algae based TKTD models so they are reliable, standardized, and ecologically meaningful tools for contemporary chemical risk assessments.

## Methods

### Data Sources and Search Strategy

Studies were sourced from the *Web of Science* and *Google Scholar* (search conducted June 2026). The keywords included were ((diatom* OR cyanobacteria* OR “blue-green algae” OR “blue green algae” OR “green algae” OR “unicellular algae” OR phytoplankton* OR microalga* OR algal* OR algae*) AND (toxicokinetic* OR toxicodynamic* OR “toxicokinetic-toxicodynamic” OR “toxicokinetic toxicodynamic” OR “population model” OR “pulse exposure”)). The studies were then reviewed and filtered for relevance based on the selection criteria. For further description of the systematic review, refer to the PRISMA^18^ diagram in the SI (Figure S1).

### Selection Criteria and Data Structure

This review included studies that used TKTD models for freshwater planktonic microalgae, utilizing laboratory derived data for calibration (parametrization), and carried out a validation step. In this context, model parameters refer to quantitative values that define model behavior and biological processes. TK parameters describe how chemicals are absorbed, distributed, metabolized, and excreted (ADME) by an organism. TD parameters outline processes at the target site, including damage and recovery. Calibration involves adjusting model parameters so that the observations fit. Validation tests a model’s accuracy and predictive ability by comparing its outputs to data not used during calibration. To characterize the extent of independent model validation, studies were assigned a grouping of A, B, or C, based on the scope and independence of the data used for validation. (A) calibration only—model performance was validated using the same data employed for parameter estimation, with goodness-of-fit (GoF) metrics or visual agreement assessments, but without testing predictive performance using independent data. (B) independent validation within the same context—model predictions were tested against independent data sets, scenarios, or conditions that differed from the calibration data set but remained within the same biological and experimental context (e.g., different exposure profiles for the same species under similar laboratory conditions). (C) independent validation beyond calibration context—model predictions were tested using independent data sets representing different biological or experimental contexts such as different exposure systems (e.g., field studies, flow-through systems), environmental conditions, or species. The grouping reflects the breadth of independent validation rather than the overall validity or regulatory suitability of a model. A model classified as group B may be considered fully adequate for applications confined to the biological and experimental context for which it was validated, whereas group C indicates that predictive performance has also been examined across a broader range of contexts. Studies assigned to group A were kept for descriptive purposes but were not considered evidence of predictive performance.

### Data Extraction

After reviewing the literature, information was collected for comparison. The details, including but not limited to the chemical name, classification, MoA using the 2026 Herbicide Resistance Action Committee (HRAC) classifications,^19^ algae species, the biological end point of interest, growth factors used for calibration, the exposure scenario being modeled, the model type, the model fit, validation grouping, and validation method, were extracted.

## Results

Twelve papers on TKTD modeling of freshwater microalgae, ranging from the years 2012 to 2023, met the selection criteria. Information on data filtering can be found in the PRISMA^18^ diagram in the SI. These studies applied mechanistic models to address specific research questions. The main objectives of the studies included evaluating the effects of varying exposure durations and recovery periods, exploring different compartmentalization strategies (e.g., separating TK and TD), calibrating models under diverse environmental conditions (e.g., temperature, light intensity, media renewal rates), assessing chemicals with distinct MoA, and investigating interspecies competition (Table 1).

**Table 1:** Summary of the Chemical/Species/Growth Factor Combinations Tested and the Models Used, Divided into Groups of Similar Chemical Mode of Action Using the Herbicide Resistance Action Committee (HRAC) Classification for the Herbicides^a^

		Biological		Growth factors				Model		
References	Chemical	Algae species	Additional end points	Temperature (°C)	Nutrients (N, P mM)	Light (μmol m^–2^ s^–1^)	Exposure scenario	Type	Fit	Validation grouping
	Herbicides: Sulfonylureas [Group: 2]									
Nagai, 2014	Bensulfuron-methyl	RS		23 ± 1	0.28, –	50	Long-term exposure 100 d	TKTD	Can predict population with good fit, but TK is necessary	A, SS
								One compartment first-order kinetic population growth, with TK component		
	Herbicides: Ureas [Group: 5]									
Weber et al., 2012	Isoproturon	DS		27	0.28, 9.2	120	FT, MP, 80 d	Direct effect	Can predict both the level of growth inhibition and the rate of recovery of the population	C, FT
								TD population growth model with growth factors (no explicit TK); Full SAM-X		
Weber et al., 2012,		RS		28	0.28, 9.2	120	FT, MP, 80 d	Direct effect	Can predict both the level of growth inhibition and the rate of recovery of the population	C, FT
								TD population growth model with growth factors (no explicit TK); Full SAM-X		
Rendal et al., 2023,				28	0.28, 9.2	120	Long-term exposure 500 d	TKTD	Can be used in a moving time window	C, FT^b^
								TD population growth model with growth factors (optional TK); Full/simplified SAM-X		
Copin and Chèvre, 2015				25	0.28, 9.2	30	SS, MP, 98 h	Direct effect	The model from Copin et al. 2015 has a good fit, instant recovery	B, SS
Copin and Chèvre, 2018		SV-RS		25	0.28, 9.2	30	SS, SP, 54–73 h	Direct effect	Can predict the population of one species in coculture	B
Copin et al., 2015		SV		25	0.28, 9.2	70	SS, MP, 50–88 h	Direct effect	The model from Copin et al. 2015 has a good fit, instant recovery	B, SS
Vogs et al., 2015			ADBE	28 ± 0.5	8, 4 ^20^	370 ^20^	SP, 4 h	TK	Can predict chemical kinetics	A
								One compartment TK		
Vogs and Altenburger, 2016			ADBE, AOP	28 ± 0.5	8, 4 ^20^	370 ^20^	SP, 24 h	Extended mechanistic TKTD	Can predict population with a good fit	A
								AOP-based effects		
Copin and Chèvre, 2015	Diuron	SV		25	0.28, 9.2	30	SS, MP, 98 h	Direct effect	The model from Copin et al. 2015 has a good fit, instant recovery	B, SS
Weber et al., 2019	Chlorotoluron	RS		24	0.28, 9.2	120	FT, MP, 91/94 d	Direct effect	Can predict both the level of growth inhibition and the rate of recovery of the population	C, FT
								TD population growth model with growth factors (no explicit TK); Full SAM-X		
Rendal et al., 2023				24	0.28, 9.2	120	Long-term exposure 500 d	TKTD	Can be used in a moving time window	C, FT^b^
								TD population growth model with growth factors (optional TK); Full/simplified SAM-X		
	Herbicides: Triazines [Group: 5]									
Copin and Chèvre, 2015	Atrazine	SV		25	0.28, 9.2	30	SS, MP, 48 h	Direct effect	Can predict the population of one species in coculture	B, SS
	Herbicides: *N*-Phenyl heterocycles [Group: 12]									
Vogs et al., 2013	Norflurazon	SV	Cell volume, C_int_, AOP	28 ± 0.5	8, 4 ^20^	370	SP, 24 h	Extended mechanistic TKTD	Effect propagation describes the delay in growth inhibition well.	A
								Effect propagation		
	Herbicides: *N*-Phenyl-imides [Group: 14]									
Nagai, 2014	Pentoxazone	RS		23 ± 1	0.28, −	50	Long-term exposure 100 d	TKTD	Can predict population with a good fit, but TK is necessary	A, SS
								One compartment first-order kinetic population growth, with TK component		
	Herbicides: α-Chloroacetamides [Group: 15]									
Nagai, 2014	Pretilachlor	RS		23 ± 1	0.28, −	50	Long-term exposure 100 d	TKTD	Can predict population with a good fit, but TK is necessary	A, SS
								One compartment first-order kinetic population growth, with TK component		
Vogs and Altenburger, 2016	Metazachlor	SV	ADBE, AOP	28 ± 0.5	8, 4 ^20^	370 ^20^	SP, 24 h	Extended mechanistic TKTD	Can predict population with a good fit	A
								AOP-based effects		
Vogs et al., 2015			ADBE	28 ± 0.5	8, 4 ^20^	370 ^20^	SP, 4 h	TK	Can predict chemical kinetics	A
								One compartment TK		
Copin et al., 2016	S-metolachlor	SV		25	0.28, 9.2	30	SS, MP, 117 h	Direct effect	Can predict populations when there is a delayed effect	B, SS
	Herbicides: Pyridiniums [Group: 22]									
Vogs and Altenburger, 2016	Paraquat	SV	ADBE, AOP	28 ± 0.5	8, 4 ^20^	370 ^20^	SP, 24 h	Extended mechanistic TKTD	Can predict population with a good fit	A
								AOP-based effects		
Vogs et al., 2015			ADBE	28 ± 0.5	8, 4 ^20^	370 ^20^	SP, 4 h	TK	Can predict chemical kinetics	A
								One compartment TK		
	Herbicides: Other Unknown MoA									
Nagai, 2014	Quinoclamine	RS		23 ± 1	0.28, –	50	Long-term exposure 100 d	TKTD	Can predict population with a good fit, but TK is necessary	A, SS
								One compartment first-order kinetic population growth, with TK component		
	Biocide									
Vogs and Altenburger, 2016	Irgarol 1051	SV	ADBE, AOP	28 ± 0.5	8, 4 ^20^	370 ^20^	SP, 24 h	Extended mechanistic TKTD	Can predict population with a good fit	A
								AOP-based effects		
Vogs et al., 2015			ADBE	28 ± 0.5	8, 4 ^20^	370 ^20^	SP, 4 h	TK	Can predict chemical kinetics	A
								One compartment TK		
Vogs and Altenburger, 2016	Triclosan	SV	ADBE, AOP	28 ± 0.5	8, 4 ^20^	370 ^20^	SP, 24 h	Extended mechanistic TKTD	Can predict population with a good fit	A
								AOP-based effects		
Vogs et al., 2013			Cell volume, C_int_, AOP	28 ± 0.5	8, 4 ^20^	370 ^20^	SP, 24 h	Extended mechanistic TKTD	Effect propagation describes the delay in growth inhibition well.	A
								Effect propagation		
Vogs et al., 2015			ADBE	28 ± 0.5	8, 4 ^20^	370 ^20^	SP, 6 h	TK	Can predict chemical kinetics	A
								One compartment TK		
Industrial										
Vogs and Altenburger, 2016	*N*-phenyl-2-naphthylamine (PNA)	SV	ADBE, AOP	28 ± 0.5	8, 4 ^20^	370 ^20^	SP, 24 h	Extended mechanistic TKTD	Can predict population with a good fit	A
								AOP-based effects		
Vogs et al., 2013			Cell volume, C_int_, AOP	28 ± 0.5	8, 4 ^20^	370 ^20^	SP, 24 h	Extended mechanistic TKTD	Effect propagation describes the delay in growth inhibition well.	A
								Effect propagation		
Vogs et al., 2015			ADBE	28 ± 0.5	8, 4 ^20^	370 ^20^	SP, 4 h	TK	Can predict chemical kinetics	A
								One compartment TK		
	Pharmaceutical intermediate: Dichlorophenol (OECD reference compound)									
Weber, 2013	3,5-dichlorophenol	DS		23 ± 0.5	0.28, 9.2	76 ± 10	FT, MP, 72 d	Direct effect	Can predict both the level of growth inhibition and the rate of recovery of the population	C, FT
								TD population growth model with growth factors (no explicit TK); Full SAM-X		

a The biological parameters consist of the algae species (DS = *Desmodesmus subspicatus*, RS *=* *Raphidocelis subcapitata* (formerly *Pseudokirchneriella subcapitata*), SV = *Scenedesmus vacuolatus*), and monitored end points (growth rate unless otherwise stated; ADBE = absorption, distribution, metabolism, and excretion, AOP = adverse outcome pathway). Growth factors for calibration data (all preformed in static conditions and following OECD guidelines), are given together with exposure scenarios (FT = flow through, SS = semistatic, SP = Single pulse, MP = multiple pulses, length of modeled exposure (See Figure 2)), model type, model fit, and model validation data set and type of validation exposure preformed (FT = flow through, SS = semistatic).

b Means validation conducted in a previous study, as explained in the text below. Reference to original studies are given in the first column.

### Chemicals and Modes of Action

In the 12 papers, 16 chemicals were modeled. The main MoA tested were photosystem inhibitors (Group 5; *n* = 4) and lipid biosynthesis inhibitors (Group 3, *n* = 3), followed by other MoAs (*n* = 9; Table 1). Many of the papers approached the modeling from a pesticide regulation perspective, as this is currently the major regulation accepting TKTD model outputs, hence, many of the chemicals were herbicides. However, three papers focused on the regulation of other chemicals, including two biocides,^13,14^ an industrially used chemical,^13–15^ and a pharmaceutical intermediate^22^ (3,5-dichlorophenol, recommended as a reference compound^17^). Isoproturon is frequently used because it induces rapid and reversible growth inhibition, such that damage closely follows internal concentration, and recovery processes can be neglected in the modeling.^23^

### Algae Species

In the 12 papers, three species of green microalgae were tested (Table 1). The species studied were *Scenedesmus vacuolatus* (*n* = 7), followed by *Raphidocelis subcapitata* (a.k.a. *Pseudokirchneriella subcapitata*/*Selenastrum capricornutum*; *n* = 6), and *Desmodesmus subspicatus* (a.k.a. *Scenedesmus subspicatus* *n* = 2), which are all green algae. The three species differ in morphology, particularly in shape, but share similar ecological niches and growth rates, allowing them to be cultured under comparable conditions (e.g., nutrients, light, temperature). Notably, the work by Weber (2013)^22^ modeled chemical effects on another species of green algae, *Chlamydomonas terricola*, and a Cryptomonad, *Cryptomonas pyrenoidifera*, but these were not used in the flow-through validation of the model.

### End Points

The models were calibrated using growth rate (or inhibition of growth rates; EC_*x*_) derived from OECD Test Guideline 201, which is a 72 h growth inhibition test for freshwater microalgae using static exposure.^17^ Two papers used published end point data from the literature instead of their own data.^13,24^ The exposure times to measure effect concentration values varied widely, from 6 to 72 h. The methods for determining these end points also differed. One study distinguished between live and dead algae cells.^21^ In this research, cell viability was assessed using a green nucleic acid dye called SYTOX green, with the number of live and dead cells being quantified by flow cytometry. This approach allowed the incorporation of mortality and recovery dynamics into the model, represented as live and dead cell densities rather than growth rates alone. Some studies used absorbance (optical density) and fluorescence,^22–28^ while others used microscopy and hemocytometer chambers to count cells,^22–25^ or employed an electric particle counter to determine growth rates.^14,15,21^ The most common units reported were cells per liter, regardless of the method used to assess growth. It was noted that some techniques had higher error rates, but comparison studies showed that counting under a microscope provided the highest accuracy.^22,23^ Other end points included total cell number and cellular volume,^14,15^ as well as the chemical concentration at different stages of absorption, distribution, metabolism, and excretion (ADBE). Additionally, in the TK specific papers, chemical uptake and elimination were measured, so chemical concentrations in the media and cells were reported instead of nominal values.^13,21^ The steps leading to biological effects were also mapped using adverse outcome pathways (AOPs), in which a molecular initiating event is followed by key events leading to the adverse outcome.

## Experimental Designs

### Growth Factors

Environmental growth factors of the calibration data were held constant within individual experiments but varied among studies. Temperature ranged from 23–28 °C depending on species and study design. Nutrient concentrations were typically reported as nominal starting values based on medium composition. In the studies that did not use flow through systems, nutrients were assumed to be in excess of growth limiting concentrations throughout the study. Light intensity was often reported in μE m^–2^ s^–1^ or the equivalent μmol m^–2^ s^–1^ Photosynthetic Active Radiation (PAR), and was generally held constant apart from the Vogs et al. works, where a 14:10 h light:dark rhythm was used.^13–15^ For the papers with external validation, where the models were tested against independent data sets distinct from those used for calibration, the models showed a good fit of control growth despite slight deviations in growth factors from the calibration.^22–25^

### Exposure Scenarios

The reviewed papers used a variety of exposure scenarios depending on their research objectives (Figure 2). All the papers used standardized static exposure tests, consistent with OECD Test Guideline 201 ^17^ for model parametrization. In this setup, algae are grown in batch cultures, typically in Erlenmeyer flasks exposed to an initial chemical concentration over a fixed period, and exposure over the experiment follows the dissipation rate of the chemical under the given test conditions. Adaptations to the OECD setup have been made for high throughput testing.^29^ In these setups, 96-well microplates are placed on top of an LED panel, and algae density is measured at regular intervals in a spectrophotometer. Both methods for generating parametrization data have their pros and cons. The flasks allow for more agitation and volume, but can be restricted by the setup as space quickly becomes limiting in most laboratories, reducing replication and concentrations. Microplates are difficult to agitate, and the algae can settle to the bottom of the wells, causing unequal chemical exposure and density dependent growth increases. This has also been reflected in higher variability between replicates. On the positive side, this method allows for the testing of many treatments and replicates simultaneously, more frequent spectrometry measurements, using smaller test volumes, and often shorter exposure times, thereby reducing evaporation and CO_2_ limitation issues.^29^ Apart from static exposures, semistatic exposure were used (Figure 2) which involves spinning down the algae and renewing either with the same chemical concentration or with clean media. The latter is called pulse-exposures, and can either be single pulse (SP), or multiple pulses (MP) exposures with recovery periods of different durations in between. The flow-through or chemostat setup also allows for the chemical concentration to be held constant (e.g., FOCUS 1, 3, or 20 d) or exposed for long-term durations (exposure lasting 100–500 d; Table 1) since the medium containing the chemical is continually renewed into the test chamber using a pump system.

**Figure 2:**
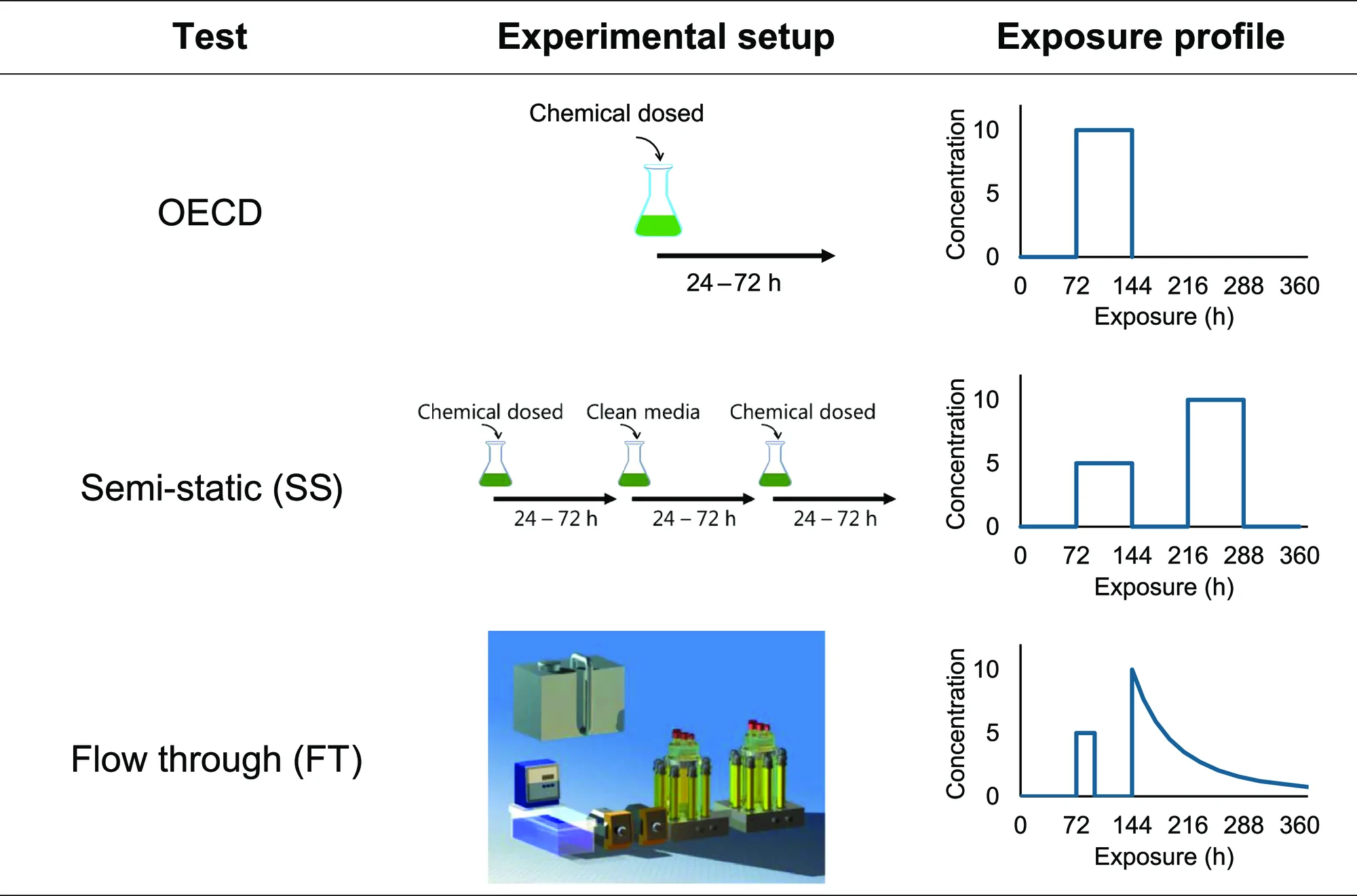
Description of laboratory test setups used to calibrate and validate the models, and the type of chemical exposure profiles that can be tested. Row 1 describes the recommended test setup from the OECD guideline 201 for growth inhibition of algae. In these tests, algae are typically grown in Erlenmeyer flasks and are exposed to a single pulse of chemicals for 24–72 h. Row 2 describes a method using a semistatic exposure where OECD setups are used and exposed algae are transferred to clean media to allow for a recovery phase. This can be repeated over different time intervals to simulate multiple pulses and variable exposure and recovery times. Row 3 describes flow-through (chemostat) setup illustrated in Weber (2013),^22^ used to validate the SAM-X model. In this setup, medium (with or without toxicants) is continuously added and is removed with the outflow from the system, maintaining control populations at a constant density determined by the growth factors (e.g., light, temperature, and nutrient levels) and the flow rate.

### Classification of Algal TKTD Models

The reviewed studies range from simple TD models based on direct effects of external exposures to mechanistic TKTD frameworks with explicit TK and damage compartments. An overview of the different model components used in each study has been summarized in Table 2 and in Figure S2. Based on their assumptions and level of mechanistic detail, the reviewed studies can be grouped into three classes, beginning with direct effect models that relate biological responses directly to external exposure.

**Table 2:** Comparison of the Major Algae TKTD Model Classes Identified in the Literature

Model type	Main assumptions	Data requirements	Advantages	Limitations	Applications
TK models	Describes the uptake and elimination of a compound without consideration of a growth model; Assumes first-order uptake and elimination processes within a single compartment	Time-resolved chemical concentrations in exposure medium and organism, biomass, uptake/elimination kinetics	Provides quantitative uptake and elimination parameters, provides equilibrium time	Does not describe toxic effects or growth dynamics; one-compartment models may miss other processes	Provides TK parameters for subsequent TKTD modeling
Direct effect (TD) models	External concentration directly drives effects; no explicit internal concentration	OECD growth inhibition and pulse exposure data	Simple, transparent, low parameter demand	Cannot explicitly represent internal exposure processes	Prediction of algal growth responses under externally driven exposure scenarios (constant and pulse); screening and regulatory applications (e.g., Tier 2C)
TKTD models	Internal concentration approximated by rapid equilibrium or first-order uptake/elimination	Exposure time series, uptake/elimination kinetics, and growth data	Improved recovery predictions with moderate complexity	Limited representation of mechanistic processes	Prediction of algal growth under time-variable exposure, including pulse and sequential exposures; evaluation of uptake-driven delays in toxicity; regulatory applications where recovery dynamics are important (e.g., Tier 2C)
Extended mechanistic TKTD models	Internal concentrations and damage propagation explicitly represented	Internal concentrations, uptake/elimination kinetics, effect time-series, mechanistic end points	Captures delayed effects, AOPs, and chemical specific processes	High complexity and data requirements	Mechanistic research and hypothesis testing

### TK Models

Vogs et al. 2015,^13^ developed a one-compartment TK to quantify bioconcentration kinetics of specifically acting and reactive chemicals. This model is a standalone TK model and does not include an algal growth model. Internal exposure is derived from time-depending depletion of chemicals in the medium. The model describes uptake (*k*_in_) and overall elimination (*k*_out_) as first-order processes and accounts for the ratio of algal biovolume to exposure volume. For these parameters, kinetic and steady state bioconcentration factors (BCF_kin_) and the time to reach 95% equilibrium (*t*_95_) were derived. The study linked the TK model to effect data by multiplying EC_50_ values with BCF_kin_ to predict internal effect concentrations. The model components included hydrophobicity specific driven uptake, elimination, biotransformation, pH dependent speciation, and ion-trapping, and extrapolation for external effect concentrations to internal effect concentrations.

### Direct Effect (TD) Models

Weber et al. (2012,[Bibr ref23] 2013,[Bibr ref22] 2019 ^25^), developed the first model called SAM-X, calibrated using a static exposure setup measuring effects on algae density in response to variable chemical concentrations and validated over extended periods using the flow-through system and pulsed exposure (Figure 2). The SAM-X model assumes rapid equilibrium between external chemical concentration and internal cellular concentrations. The end point of relative growth rate (RGR) is then based on the external concentration (*C*_ext_), phosphorus or other nutrient content of media (P), irradiance (Irr), temperature (T), algae dilution, and algae death rate, together summing “loss rates”. All the environmental factors were time-dependent functions. Therefore, these variables, such as temperature, can fluctuate during the simulation, and the relative growth rate is dynamically updated at each time step to reflect these changes.

While SAM-X incorporates environmental drivers affecting algal growth, later direct effect models focused on refining the description of responses to fluctuating exposure profiles without introducing explicit TK processes. Copin et al. (2015,[Bibr ref27] 2015,[Bibr ref26] 2016,[Bibr ref28] 2018 ^30^) developed a series of TD models specifically designed to predict algal responses to pulse exposure scenarios based on external exposure concentrations. In the initial studies, the models assumed negligible TK processes and treated the effect as directly linked to external concentrations, as well as assuming immediate effect and recovery for PSII inhibitors, a very special group of herbicides affecting photosynthetic electron transport causing rapid effects on photosynthetic efficiency and growth. Predictions were validated against semistatic experiments involving centrifugation and renewal of clean medium.^26,27^ They found that the duration of peaks, particularly long pulses, was more influential on cell density than short high peaks, where fast recovery was seen. The model was later adapted to include time dependent effects and delayed recovery for S-metolachlor, introducing additional parameters (*t*_effect_, delay to onset of effects and *t*_delay_, delay in recovery) to account for the delay in effect and recovery.^28^ In this model, they observed post exposure sensitivity in multiple pulse scenarios. Finally, the framework was extended to coculture experiments to account for interspecific interactions between two algal species.^30^ In these studies, the population dynamics were based on growth inhibition without any growth factor influences. They found that growth inhibition, as species-specific EC_50s_ and cell-density inhibition, was lower in coculture than in monoculture, with 2.5 fold higher EC_50s_ for *S. vacuolatus* and 2.6 fold higher for *P. subcapitata*. Interspecific interactions, possibly due to allelopathy or competition, we suggested as factors changing algal sensitivity.

### TKTD Models

The next step in model development retained the direct relationship between external and internal exposure while introducing an optional TK component for regulatory applications. Rendal et al. (2023)^24^ demonstrated how the SAM-X model could be used in a regulatory application for Tier 2C risk assessment. The authors simplified SAM-X by removing the dynamic parameters for temperature, light intensity, and nutrient availability (internal/external P) affecting growth rate. They used moving time windows across time-variable FOCUS exposure profiles.^1^ Each simulation maintained constant laboratory conditions, with the only deviation being the replacement of constant exposure with time variable concentrations. This approach generates thousands of overlapping 72 h simulations along exposure profiles extending upward of 500 d. In addition to the EC_50_, the multiplication factor required to cause 50% growth inhibition (EP_50_) for a specific profile was calculated, which is used for Tier 2C risk decisions.^16^

Beyond its regulatory adaptation, subsequent studies increased mechanistic detail by incorporating TK processes and more realistic descriptions of algal growth dynamics. Nagai (2014)^21^ distinguished between algistatic effects, defined as growth inhibition without chemical inflicted cell death, and algicidal effects, the quantification of dead cells beyond the normal mortality included in the cell cycle. They also implemented a TK function where uptake and elimination rate constants are included with *C*_effect_ and *C*_water_ (equivalent to *C*_ext_) parameters, and a biomass growth dilution factor (*C*_effectμ_). The biomass dilution factor changes the internal concentration of the chemical as cells grow. They also included the end point for biomass loss (BioL), which quantified the cumulative reduction in biomass relative to the control over the entire exposure and recovery period, rather than the effect associated with an individual pulse in a multiple pulse scenario.

### Extended Mechanistic TKTD Models

The most mechanistically detailed models explicitly separated TK and TD processes, allowing internal concentrations, damage progression, and recovery to be represented independently. Vogs et al. (2013 ^15^ and 2016 ^14^) demonstrated that internal algae concentrations typically reached steady state within minutes, whereas growth inhibition developed cumulatively over several hours, therefore, the authors implemented a cell-cycle-based TKTD model. The model links ambient concentration to internal concentration and initiates damage above a no effect concentration. TD processes were represented by an injury rate, a recovery rate, and an explicit effect progression rate across sequential damage compartments. This captures reversible damage and delayed propagation toward end points such as cell volume. The overall model structure resembles the GUTS model in that it separates TK and TD processes through a damage variable.^31^ However, unlike classical GUTS, where damage is linked to survival outcomes through either individual tolerance (IT) or stochastic death (SD) assumptions, the Vogs et al. model represents damage propagation through sequential biological processes, resulting in continuous sublethal end points. In the later study, the differences in effect progression rates were described by adverse outcome pathways (AOPs) linking chemical exposure to molecular interactions (injury and recovery rates), progressive damage (biochemical effects, physiological effects, and phenotypic effects), and ultimately leading to growth inhibition.

### Mathematical Structure of Major Model Types

The models reviewed in the study have similar component structures depending on their complexity. Figure S2 illustrates these components conceptually. The mathematical formulations describing these components can be summarized into three main equations. The simplest models assume that external concentration directly determines growth inhibition and do not explicitly represent internal concentrations. Direct effect models of population growth can be described by

$$\frac{\mathrm{dN}}{\mathrm{dt}}=N\cdot \left(\mu \cdot f\left(x\right)\cdot f\left(C\right)-m\right)$$

Where *N* is the algae density, μ is the growth rate, and *f*(*C*) describes the effect of a chemical stressor on growth. Such formulations were used in direct effect models and the SAM-X framework, where rapid equilibrium between external and internal concentrations is assumed, and no explicit TK component is included.^21–28,30^ In the SAM-X models, additional environmental forcings are added to the model represented by *f*(*x*) and can be factors such as light, temperature, and nutrient dependence.^22,23,25^ The algae loss rate through natural mortality or by dilution can also be included, represented by *m*.^1–4^

More mechanistic approaches do not assume instantaneous equilibrium, but introduce internal concentrations as a state variable to describe the toxicokinetic compartment. Most reviewed studies described uptake and elimination using first-order kinetics to predict the internal effect concentrations (*C*_effect_)­

$$\frac{dC_{effect}}{\mathrm{dt}}=k_{in}C_{ext}-k_{out}C_{\mathrm{int}}$$

Where *k*_in_ and *k*_out_ represent uptake and elimination rate constants, respectively. Some formulations further accounted for dilution by growth,^21^ biotransformation,^13^ and ion trapping.^13^ These extensions allow internal exposure to differ from external concentrations and improve predictions when uptake or elimination processes are rate-limiting.

To describe toxicodynamic processes, a damage variable and/or effect propagation can be included. In these models, internal concentrations do not immediately translate into growth inhibition. Instead, toxic effects accumulate and may be partially repaired or recovered over time. Damage dynamics can be represented as

$$\frac{\mathrm{dD}}{\mathrm{dt}}=k_{d}\left(C_{\mathrm{int}}-NEC\right)-k_{r}D$$

And population growth then becomes

$$\frac{\mathrm{dN}}{\mathrm{dt}}=N\left(\mu \cdot f\left(x\right)\cdot \left(1-D\right)-m\right)$$

Where *D* is accumulated damage, NEC is the no-effect concentration below which the algae show no visible damage, and *k*_d_ and *k*_r_ are the injury and recovery rate constants. Effect propagation can also be included, where multiple stages of damage corresponding to successive biological growth responses to be captured can improve the description of recovery and pulsed exposures.^14,15^

### Model Validation

Across all tested compounds and exposure scenarios, the reviewed models demonstrated overall good to strong performance in describing both growth inhibition and recovery dynamics on validation data sets, although the criteria used to assess model performance varied between studies. Most implementations validated fit through visual agreement between predicted and observed growth rates.^22–25^ Quantitative validation was less common and included statistical analysis using the sum of squared residuals^21^ and mean absolute error.^13–15^ The works by Copin et al. considered a successful model fit if the observed data fell within the first and third quartiles of the box plots.^26–28,30^ Most implementations increased model performance with the inclusion of a TK component.

One of the selection criteria for the reviewed literature was that model parameters were from laboratory collected data sets, and that the model predictions were validated against independent observations to some extent. Independent validation varied among the reviewed studies (Table 1). Four studies were classified as group A (calibration only), four as group B (independent validation within the calibration context), and 4 group C (cross context validation). Group A studies validated model performance using the same data sets used for calibration.^13,15,21^ Nagai 2014, for example, calibrated the model using static OECD 201 experiments and performed long-term simulations without independent validation. Group B studies validated predictions using independent data sets collected within the same biological and experimental context. A common approach was calibration using standard static OECD 201 experiments followed by validation against semistatic or pulse exposure experiments for the same species. For example, Copin et al. 2015 calibrated the model using static OECD 201 data and validated it under semistatic exposure conditions. Semistatic systems offer high reproducibility and comparability with standardized regulatory tests, but do not necessarily account for environmental processes such as continuous nutrient input or biomass loss. In the TK study by Vogs et al. (2015)^13^ static exposure systems were also used to derive uptake/elimination rates by measuring concentration depletion over time. These data sets were then integrated into TKTD frameworks.^14,15^

Group C studies validated predictive performance beyond the calibration context. Most commonly, models calibrated under static laboratory conditions were tested using flow-through exposure systems or other independent experimental settings with fixed TKTD parameters for validation. The flow-through systems were used in several studies^23,25,22,24^ to test model predictions under more environmentally realistic conditions by introducing continuous medium renewal, controlled dilution rates, and dynamic exposure profiles. These systems enabled validation of model predictions for long-term population dynamics, fluctuating chemical concentrations, and multigenerational recovery while maintaining stable environmental conditions. Since model calibration was based on standard OECD tests whereas validation was performed in an independent flow-through exposure system, these studies represented validation beyond the original calibration context. Although flow-through systems better mimic freshwater environments such as streams and drainage ditches, they require greater technical complexity and are less directly comparable with standardized OECD batch tests. As highlighted by Rendal et al. (2023),^24^ semistatic setups more closely resemble the conditions of the OECD 201 test and are therefore directly aligned with Tier 2C risk assessment scenarios.

## Discussion

### Model Complexity versus Purpose

Mechanistic models span different levels of complexity, which presents a challenge in TKTD modeling in balancing mechanistic realism with practical applicability. While increasing complexity may improve biological interpretability and predictive scope, this often comes at the cost of greater parameter uncertainty, reduced transparency, and more limited regulatory application. The key discussion point is therefore not which model is “best”, but which level of complexity is appropriate for the intended scientific or regulatory question. The principal characteristics of the major algae TKTD models identified in this review are summarized in Table 2.

Figure 3 provides a generalized workflow for selecting an appropriate model complexity to align with study objectives and system characteristics.

**Figure 3:**
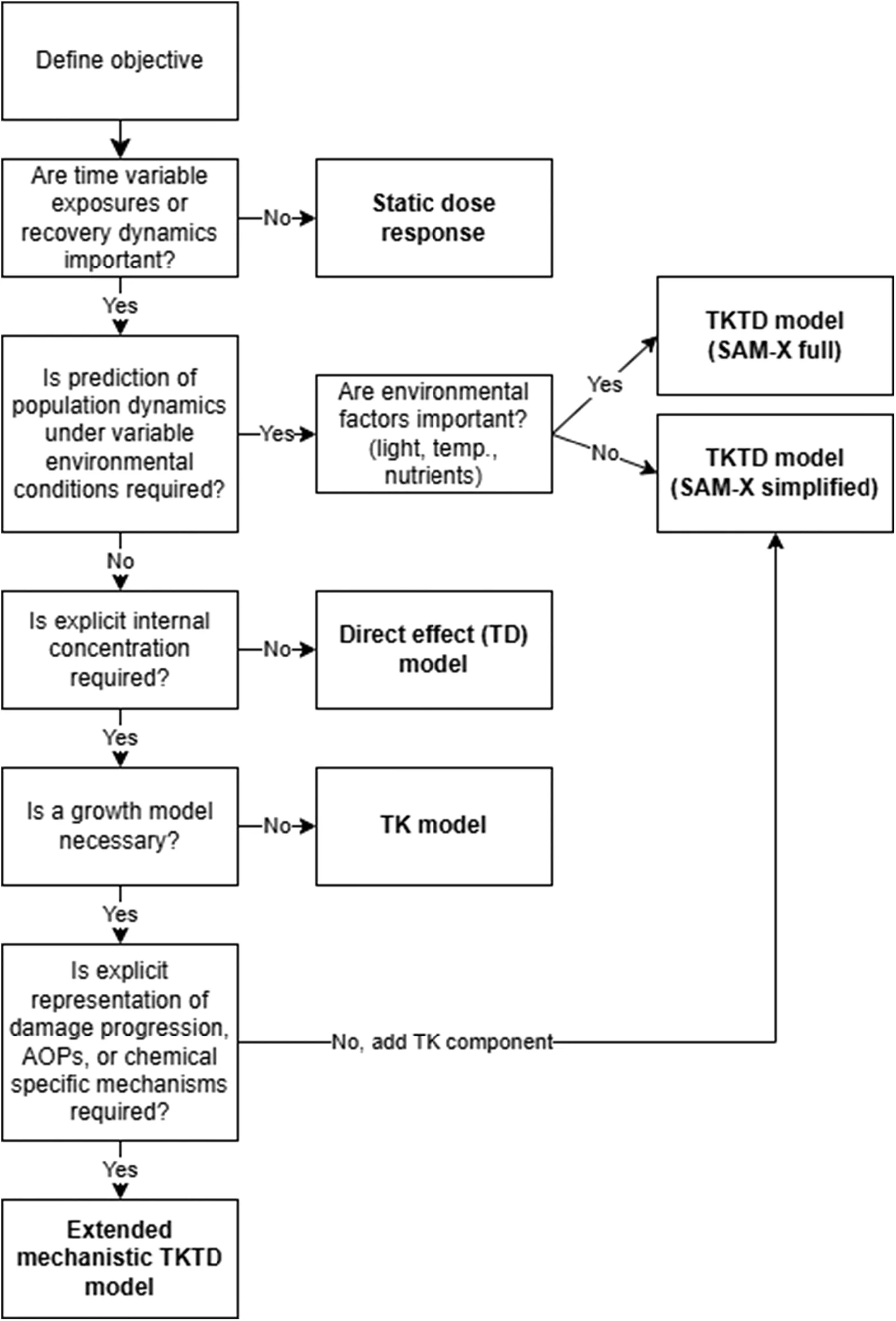
Decision tree for selecting algae TKTD model complexity based on study objectives and system characteristics. The framework follows the principle of selecting the simplest model capable of adequately describing the observed system behavior.

When deciding what is required of the model, the first thing that separates the model choice is the purpose of the application. In the case of risk assessment, using the simplified SAM-X model and increasing complexity when it fails seems to be the most suitable model for risk assessment at the moment.^16,24^ Compared with simpler growth inhibition models, SAM-X offers improved ecological realism by explicitly incorporating fluctuating growth factors. The benefit of this additional complexity is improved applicability to environmentally variable exposure profiles and long-term simulations.^24^ However, this also increases calibration requirements and may reduce transferability when site-specific environmental data are unavailable. Outside regulatory applications, model complexity can be viewed as progressing from static dose response models through direct effect and delayed effect TD models to intermediate and fully mechanistic TKTD frameworks. Each level introduces additional parameters, thereby increasing predictive application by also increasing data requirements.

Direct effect models (TD) are characterized by low parameter demands and have been useful for predicting pulse exposure scenarios. The TK models are standalone models to describe the uptake and elimination of compounds, without the inclusion of a growth model. TKTD model approaches introduce first-order uptake and elimination processes and distinguish between reversible growth inhibition and mortality, this improves predictions of recovery dynamics. Extended mechanistic TKTD frameworks fully incorporate damage progression and AOPs, representing delayed toxicity and chemical specific processes. These approaches are currently better suited for mechanistic research than routine regulatory applications. Ultimately, model complexity should be driven by the research question, and models should be no more complex than needed while retaining ecological relevance.^32^

### When is a TK Component Necessary?

As summarized above, a central question emerging from this review is whether explicit TK representation is always required for robust algal TKTD modeling. The absence of a TK component has prompted debate about whether SAM-X qualifies as a true TKTD model.^16^ The reviewed papers, however, demonstrate that the necessity of a TK component depends on chemical properties, exposure dynamics, and intended modeling objectives, with the importance of a TK compartment diminishing as modeling scenarios expand in exposure time. Currently, the range of studied chemicals remains narrow, making general compound specific guidelines more difficult. The current studies are strongly dominated by rapidly acting PSII inhibitors, which may bias conclusions toward frameworks that omit explicit TK processes. Several studies exist comparing toxicities of different chemicals based on static exposure and 48–72 h EC50 values.^33,34^ In a study where 36 pesticides of differing MoA were tested on algae, herbicides that disrupt cell division (HRAC, Group 2), target chloroplasts (Group 5 and 6), and inhibit root or shoot growth (Group 3 and Group 15) have the highest toxicity.^34^ However, these comparative reviews are generally based on static EC_50_ values and do not include recovery dynamics, delayed effects, or differences in reversibility.

Copin et al. (2016)^28^ demonstrated that some herbicides exhibit postexposure sensitivity under multiple pulse scenarios, indicating external concentrations alone may not fully explain observed dynamics. Additionally, Vogs and Altenburger (2016)^14^ tested the hydrophobicity of compounds, expressed as octanol–water partitioning coefficient log *K*_ow_ (ranging from −4.5 to 4.8 in the chemicals studied), and their influence on uptake and elimination rates. Compounds with a log* K*_ow_ > 4 had lower elimination rate constants, whereas less hydrophobic chemicals had higher elimination rates, as would be expected. Uptake rates, however, did not correlate directly with hydrophobicity, particularly when biotransformation, ion-trapping, or intracellular pH influenced chemical fate. Explicit TK components may therefore be necessary for chemicals with different physicochemical properties from those currently tested to avoid underestimating recovery times.

The reviewed TK studies also highlight the importance of chemical specific processes, such as pH dependent speciation, membrane affinity,^35^ sorption,^36^ and biotransformation.^13^ Ionizable and reactive chemicals may undergo ion-trapping, altered membrane permeability, or rapid intracellular transformation processes, which cannot be captured by assuming equilibrium with external concentrations. Vogs et al. (2015)^13^ suggested that bioavailability may also be altered by medium conditions,^3,37^ sorption to particles, or interactions with extracellular substances,^38,39^ as is always the case in natural waters, but rarely tested in experiments. These processes introduce variability in internal exposure and are not predictable from external total concentration profiles alone. In these cases, a TK component is needed for mechanistic understanding.

The inclusion of a TK component should be guided by the processes underlying observed effects rather than by model complexity alone. The TK component is most critical when internal concentrations or damage dynamics drive observed effects, for example, in cases of effects persisting after external exposure has ceased. In such cases, TK representation improved predictions of recovery and cumulative effects under pulsed exposure scenarios. Overall, the decision to omit or include TK components should be based on whether internal concentration or damage dynamics are rate limiting for the observed response.

### Principles for Biological Representation

Beyond TK components, the biological representation of effect processes strongly influences model realism and predictive capability. In the reviewed studies, the level of biological detail directly determines which ecological questions a model can meaningfully address. Most standardized protocols rely on biomass or yield (total cell number at a fixed time point), whereas specific growth rate has been proposed as a theoretically more robust alternative. It has been shown that EC_*x*_ values from growth rate are less dependent on test system parameters (e.g., initial density and exposure duration) than those based on yield.^40^ While growth rate can in principle be derived from biomass measurements over time, it is not always explicitly used as the primary end point in experimental analysis or model calibration. Therefore, reliance on biomass-based inhibition alone may miss subtle effects, particularly when sublethal stress alters cell division rates without reducing final cell density. When more information is required to improve ecological prediction, incorporating growth rate in addition to biomass end points provides more sensitive and mechanistically informative assessments of chemical stress.

Molecular responses often occur at lower exposure concentrations and earlier time-points than traditional end points. Using transcriptomic,^41^ proteomic,^42^ and metabolomic^43^ approaches provides powerful tools for identifying sensitive and early warning indicators of toxic stress in algae. These methods enable mechanistic insight into disrupted pathways related to energy metabolism, photosynthesis, oxidative stress, and cellular homeostasis, thereby improving understanding of TD processes.^8^ Incorporating such sublethal and mechanistic end points into TD assessments can support the identification of key processes that ultimately affect growth, reproduction, and population dynamics, therefore strengthening the link between molecular level responses and ecologically relevant outcomes. This improves the interpretation of toxic effects beyond growth inhibition alone.

Overall, the reviewed studies demonstrate that increasing biological detail enhances mechanistic interpretability but also increases data and parameter requirements. As mentioned previously, for regulatory screening focused on growth inhibition under standardized conditions, simplified models may be sufficient. However, when the objective is to distinguish between underlying mechanisms (e.g., reversible vs irreversible effects), improve extrapolation across varying environmental or exposure scenarios, or link molecular processes to population level responses, a more detailed biological representation can provide additional value. In particular, the incorporation of adverse outcome pathways, although initially adding complexity, may support extrapolation across chemicals by aiding grouping based on shared molecular initiating events and key event sequences. Expanding beyond growth inhibition as a single end point would enhance mechanistic resolution and ecological interpretation.

### Regulatory Applicability: Calibration and Validation

Model credibility in regulatory contexts not only relies on model fit but also on the degree of independence between calibration and validation data sets. Calibration was generally based on standardized OECD growth inhibition tests or comparable laboratory experiments conducted under constant environmental conditions. The use of standardized test systems provides reproducible parameter estimates and allows for comparison among studies. While direct effect models can be calibrated from growth inhibition time series, models incorporating TK processes or damage progression require additional information on uptake and elimination kinetics, internal concentrations, or mechanistic end points.

The reviewed models showed variation in the independence of validation among the papers (Table 1). The review shows that relatively few algae TKTD models have been validated beyond the data sets used for their development, essentially nonvalidated models. Although several studies included independent validation, only a subset tested predictive performance under different experimental conditions. This distinction is important because agreement with calibration data demonstrates that a model can reproduce observations used for parameter estimation, whereas independent validation evaluates whether the underlying model structure generalizes to new exposure scenarios. Models validated only within the calibration data set therefore provide weaker evidence of predictive performance than those successfully tested against independent experiments. The limited number of cross context validation studies suggests that the regulatory application of algae TKTD models remains constrained by the availability of suitable validation data sets rather than by model complexity alone. Notably, the models assigned to group C were generally calibrated under standard laboratory conditions and later were validated under more realistic flow-through or time-variable exposure scenarios. Such studies provide stronger evidence that the underlying biological processes are represented robustly across different experimental conditions. In these cases, validation is based on goodness of fit rather than on the prediction of independent data sets.^31^ Predictive capability is particularly important because TKTD models can alter the interpretation of environmentally realistic exposure profiles. For example, studies by Copin et al.^26–28^ demonstrated that longer exposure durations at moderate concentrations can have a greater impact on algal biomass than short, high concentration pulses due to rapid recovery processes. This contrasts with Tier 1 assessment approaches, where short-term peak concentrations are often considered most critical. TKTD models provide a mechanistic framework to resolve such discrepancies by explicitly accounting for exposure duration, recovery dynamics, and cumulative effects, thereby improving the ecological relevance of risk assessments under realistic exposure scenarios. Overall, the review indicates that independent validation has become increasingly common in recent algae TKTD studies, particularly for models intended for regulatory applications. However, validation beyond the original experimental context remains relatively limited, highlighting the need for additional studies evaluating model transferability across exposure systems, environmental conditions, and species.

While the reviewed studies differed in the degree of independent validation, they also differed in how model performance was assessed. Model fit was assessed using a variety of approaches, including visual inspection, sum of squared residuals, mean absolute error, and quartile plots. Although these methods generally demonstrated satisfactory agreement between simulations and observations, differences in performance metrics make objective comparison between models difficult. The EFSA Scientific Opinion on TKTD models provides standardized goodness-of-fit criteria and recommendations for calibration and validation data sets.^16^ They advise not choosing any one metric for assessing model fit, but suggest including multiple criteria. Specifically, they suggest visual fit of model predictions with observed data over time and using posterior predictive check (PPC), which is the comparison of median values of the predicted and observed values, as well as the prediction uncertainty. However, direct application of some goodness-of-fit criteria to algae models is challenging because biomass increases exponentially during the test.^16^ Metrics based on absolute biomass difference may place greater weight on observations collected later in the experiment, when biomass is highest.^17^ Regulatory guidance for algal toxicity testing recommends evaluating effects on specific growth rate rather than biomass, and EFSA notes that validation criteria originally developed for GUTS require further validation before being applied to primary producer models.

Therefore, a standardized protocol for algae TKTD modeling should build on the validation framework described in the EFSA TKTD Opinion while accounting for the specific characteristics of primary producer models. This includes (I) explicit separation of calibration and validation data sets, (II) standardized model performance metrics combining qualitative and quantitative criteria, (III) fixed TKTD parameter values during validation while allowing recalibration of the physiological growth model to reproduce control growth when necessary, and (IV) validation using independent data sets appropriate for the intended application. Such a framework should ensure that the models remain reliable decision support tools for pesticide risk assessment.

## Future Challenges

### Taxonomic and Ecological Gaps

A major limitation of the current literature is that conclusions about model structure and parameter behavior are largely derived from narrow taxonomic base of green algae. This raises concerns about the generalization of current TKTD assumptions across phytoplankton groups. Of the three species that have been validated, *R. subcapitata* is generally the most sensitive in other studies comparing species,^44^ but species sensitivities often vary depending on the chemicals being tested.^45,46^ Therefore, to determine the chemical risk for algae, a selection of species with varying sensitivities to different chemical MoAs should be used to better represent the ecosystem.

From an ecosystem perspective, two groups of microalgae species are missing: diatoms and cyanobacteria. This is important because green algae are often not the dominant species, for example, diatom blooms can comprise 75% of the phytoplankton abundance, typically in the spring.^47^ In cases of nitrogen limitation, cyanobacteria can fix nitrogen from the atmosphere, giving them an advantage over other species, leading to cyanobacterial blooms.^48^ Cyanobacteria are also important to model because some cyanobacteria produce toxic substances affecting drinking water and ecosystem quality. To treat cyanobacterial blooms in urban areas, algicides are often directly applied in lakes, which can have harmful effects on other species.^49^ These ecological niches and the fact that cyanobacteria are bacteria and not eukaryotic algae suggest they likely have different sensitivities to chemicals compared to green algae.

The dominance of data on green algae in TKTD modeling is not due to a lack of toxicity data for other taxa, as diatoms and cyanobacteria are routinely included in standardized regulatory tests. However, the application of TKTD models is often driven by the need for refinement in cases where standard assessments indicated high sensitivity of effects. In this context, green algae such as *R. subcapitata* and *D. subspicatus* are frequently used due to their sensitivity and well established role in regulatory testing. In contrast, lower representation of diatoms and cyanobacteria in TKTD modeling may partly reflect a lower frequency of cases where refinement is required, rather than a lack of available data. However, species sensitivity is highly compound specific and there are examples of when green algae are not the most sensitive model organism.^50^

Growth characteristics of diatoms and cyanobacteria, however, can pose experimental challenges.^17,44^ Diatoms typically grow slowly compared to green algae, which can be a challenge for culturing and observing rapid responses to toxicants. Cyanobacteria, on the other hand, often clump and form chains, making uniform absorbance and fluorescence measurements over time more challenging. For standardized toxicity tests, the OECD guideline 201 ^17^ recommends the green algae species *R. subcapitata* and *D. subspicatus*, the diatom species *Navicula pelliculosa*, and the cyanobacteria species *Anabaena flos-aquae* and *Synechococcus leopoliensis*.^17^ These species have proven suitable for toxicity testing as they have quick reproduction and are relatively simple to cultivate. Expanding TKTD model development to better accommodate these taxa would improve freshwater algal community representation.

Beyond taxonomic representation, environmental conditions influence phytoplankton community structure in natural systems. Growth factors such as temperature, light,^51^ nutrients,^52^ trace element composition, species competition among algae, and predation have an impact on the phytoplankton dynamics in natural ecosystems.^53^ Community species composition is highly site specific and shaped by local and regional drivers, which makes generalizations difficult. The SAM-X model^22^ already incorporates key environmental drivers, including temperature dependent growth (*T*_min_, *T*_opt_, *T*_max_), light limitation (*I*_opt_), and nutrient regulation via internal cell quotas (*q*_min_, *q*_max_). In addition, the model structure allows simulation of multiple species competing for shared resources, providing a mechanistic basis for representing phytoplankton dynamics under variable environmental conditions. However, the broader application of such models across natural systems remains challenging due to the need for species specific calibration and detailed environmental input data. Given the strong influence of water chemistry, specifically nutrient availability^51–54^ on species composition, combing site specific water chemistry data with temperature gradient experiments could provide a framework for scaling locally observed effects to regional and climate change projections. Addressing these gaps is essential if TKTD models are to represent primary producer dynamics beyond a limited number of standard test species.

The difficulty of scaling models beyond standardized laboratory conditions is shown by the work of Lopez Moreire Mazacotte et al. (2023).^55^ They developed a process-based ecological model to predict primary producer dynamics in microcosms (kept under stable radiation, temperature, and nutrients) in the presence of chemical mixtures. This model represented community interactions and environmental processes but incorporated chemical effects with dose response relationships rather than a TKTD framework. While the model captured general system behavior, it showed limited ability to reproduce phytoplankton biomass patterns, particularly under assumptions of homogeneous species sensitivity. Model performance improved only when incorporating community heterogeneity, temperature-dependent responses, and time-driven tolerance development, highlighting the importance of species composition and adaptive processes in shaping phytoplankton dynamics. These findings emphasize that extending TKTD models beyond standard laboratory species and conditions will require explicit consideration of species composition, environmental heterogeneity, and ecological interactions.

### Single Chemicals to Mixtures and Communities

Natural freshwater systems are exposed to chemical mixtures and multispecies communities, yet these complexities remain unrepresented in algal TKTD models. In the environment, organisms are rarely exposed to a single chemical stressor, but a complex mixture of compounds. Therefore, testing chemical mixtures beyond pesticide formulations is an important part of higher-tiered ERA.^56,57^ Standardized toxicity tests have assessed the potential for additive, synergistic, or antagonistic effects, using models such as concentration addition or independent action.^58^ These approaches are generally considered adequate, as combined effects beyond concentration addition are rare under standard test conditions. However, such approaches rely on effect concentrations from individual chemical bioassays and therefore do not explicitly account for potential interactions at the TK level.^59^ Processes such as competitive uptake, altered biotransformation, or changes in elimination rates are typically not represented mechanistically, but should be if equilibrium between internal and external concentration is not established quickly. However, direct TK interactions among chemicals are generally considered rare at environmentally relevant low concentrations.^60^ To improve predictive power and ecological relevance, mixture modeling within TKTD frameworks should therefore incorporate a TK component allowing for interactions among multiple chemicals to influence uptake, internal distribution, and metabolism, coupled with a TD component linking internal concentrations to effects over time to generalize the model as much as possible.^57^

Ecological realism is further limited by the predominance of single-species laboratory systems. As natural systems are rarely monodominant, testing how algae species interact after chemical exposure is important. Mechanistic modeling approaches have already demonstrated the ability to capture such interactions. For example, Weber (2013)^22^ demonstrated competition between two green algae species and a green and cryptomonad (e.g., *D. subspicatus* + *P. subcapitata*, *C. terricola* + *C. pyrenoidifera*), was successfully reproduced, with model predictions closely matching observed dominance patterns and population dynamics. In these experiments, one species consistently displaced another under nutrient limited conditions, and the model accurately reproduced both the qualitative trends and quantitative time series of competition. Weber also highlighted that differences in species sensitivity to stressors could alter competitive outcomes, potentially reversing dominance patterns and leading to long-term shifts in community structure. Likewise, Copin and Chèvre (2018)^30^ models the chemical effect on a coculture using two species of green algae. Two green algae species were chosen as they had the same growth requirements for culturing, therefore, it simplified the environmental parameters to consider. In their paper, they modeled the population dynamics of a single species. These competition models should be built upon to create a community model tracking the population densities of several species occupying different niches. Further investigation should be made into the population dynamics of differing algae groups (i.e., diatom + green algae). This is important to make predictions on the food web effects and primary production.

## Implications

This review demonstrates TKTD modeling of chemical effects on freshwater microalgae has advanced considerably, yet remains limited in scope and ecological realism. To date, most published models have focused on a small number of green algal species, with model calibration and validation restricted to PSII inhibiting herbicides under controlled laboratory conditions. While these studies demonstrate the utility of TKTD approaches for time variable exposure scenarios, their applicability to natural freshwater ecosystems is constrained not by model structure alone, but by limited chemical diversity in MoA, taxonomic coverage, and challenges in integrating these models into higher level ecological contexts.

Existing models have shown that growth inhibition can be predicted with reasonable accuracy under standardized conditions, particularly within regulatory contexts where simplified frameworks such as SAM-X are designed to use OECD tests to predict time variable exposure scenarios. For such Tier 2C applications, complex toxicokinetic components may not be required, but in some scenarios, simple components can help better describe the observed effects. However, mechanistic modeling studies show internal exposure dynamics, delayed or irreversible effects, time dependent TD processes can influence growth responses, especially for chemicals with specific MoA or complex uptake and elimination processes. Therefore, model complexity should be aligned with its intended purpose.

Based on the identified knowledge gaps, the following prospects are critical for advancing TKTD modeling of freshwater microalgae:

I. Expand taxonomic coverage by developing and validating models for diatoms and cyanobacteria, in addition to green algae, to better represent natural freshwater communities.
II. Evaluate a broader range of chemical modes of action, including chemicals with delayed recovery, and compounds with varying hydrophobicity and dissociability within and across chemical classes.
III. Validate model predictions for variability in key growth factors such as light, temperature, and nutrients to represent different seasons and climate zones.
IV. Support advancement of community level modeling by integrating TKTD models as mechanistic building blocks within broader ecological frameworks that incorporate chemical mixtures and algal species interactions.
V. Apply single-species TKTD models within higher tiered experiment systems (e.g., microcosms and mesocosms) to assess their performance when integrated into more complex and ecologically relevant modeling frameworks.

## Supplementary Material



## Notes

PRISMA flow diagram describing the identification, screening, eligibility assessment, and final selection of studies included in this review (Figure S1); a figure of the reviewed literature’s TKTD model components (Figure S2) (PDF)
