# Supplementary material for: Current Strengths and Limitations of Toxicokinetic-Toxicodynamic (TKTD) Models for Freshwater Microalgae
Source: Environ Sci Technol. 2026 Oct 7;60(41):29229–44. doi: 10.1021/acs.est.6c05753 (PMC13689827; doi:10.1021/acs.est.6c05753)
Supplement: Supplementary file 1 [file es6c05753_si_001.pdf]

Supplementary information:

Current Strengths and Limitations of Toxicokinetic-Toxicodynamic (TKTD) Models for Freshwater Microalgae

The supporting information contains the PRISMA flow diagram describing the identification, screening, eligibility assessment, and final selection of studies included in this review (pg. S1). A conceptual diagram of the model components included by each of the reviewed papers provides a visual of Table 1 (pg. S2).

Emma Yenney[1*] and Nina Cedergreen[1]

[1] Department of Plant and Environmental Sciences, University of Copenhagen, Thorvaldsensvej 40, 1871 Frederiksberg C, Denmark

*Corresponding Author

Email Address: emma.yenney@plen.ku.dk

Tel. +4535333397

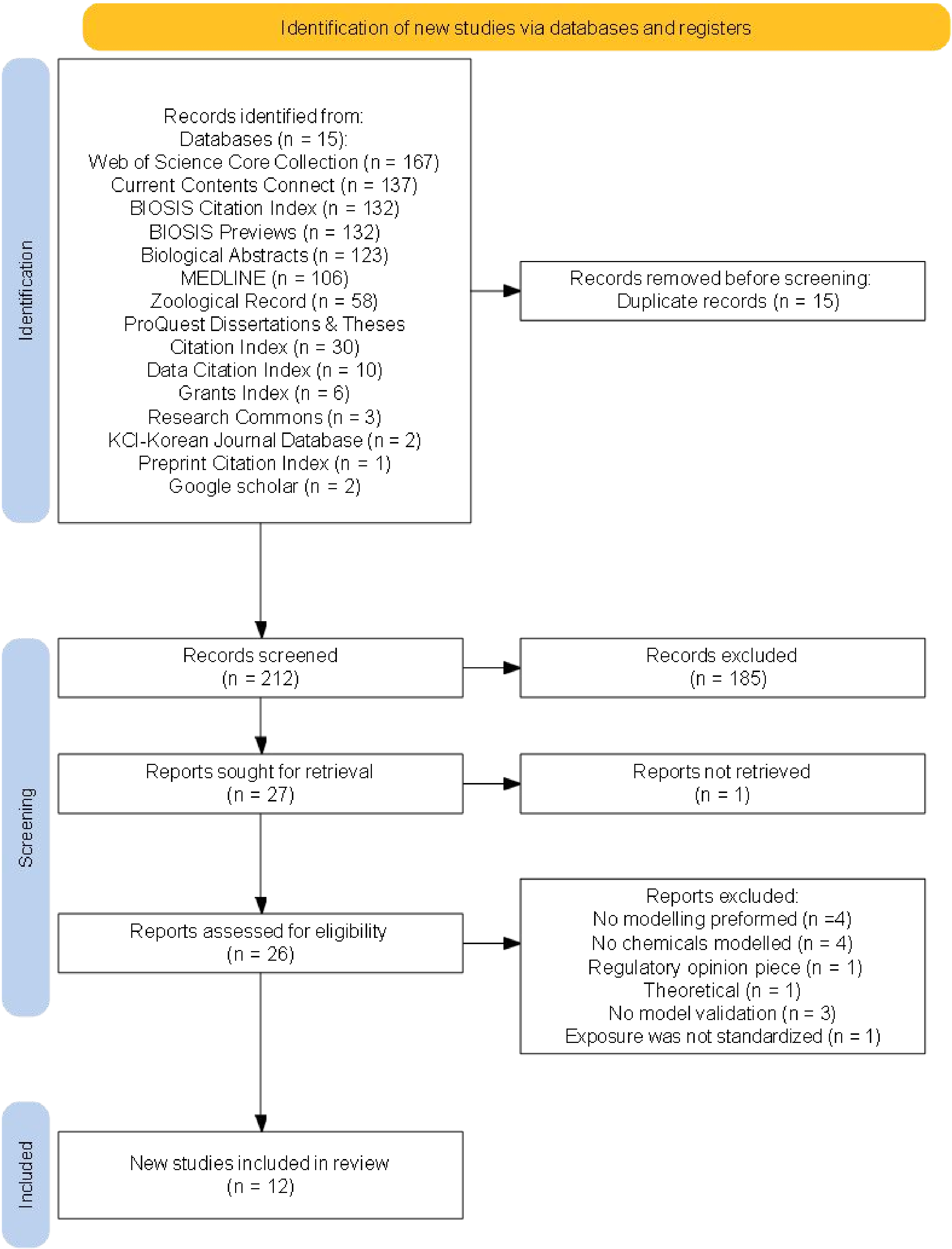

Figure S1. PRISMA diagram. Studies were sourced from the *Web of Science* and *Google Scholar* (search conducted June 2026). The keywords included were ((diatom* OR cyanobacteria* OR "blue-green algae" OR "blue green algae" OR "green algae" OR "unicellular algae" OR phytoplankton* OR microalga* OR algal* OR algae*) AND (toxicokinetic* OR toxicodynamic* OR "toxicokinetic-toxicodynamic" OR "toxicokinetic toxicodynamic" OR "population model" OR "pulse exposure")). The studies were then reviewed and filtered for relevance based on the selection criteria.

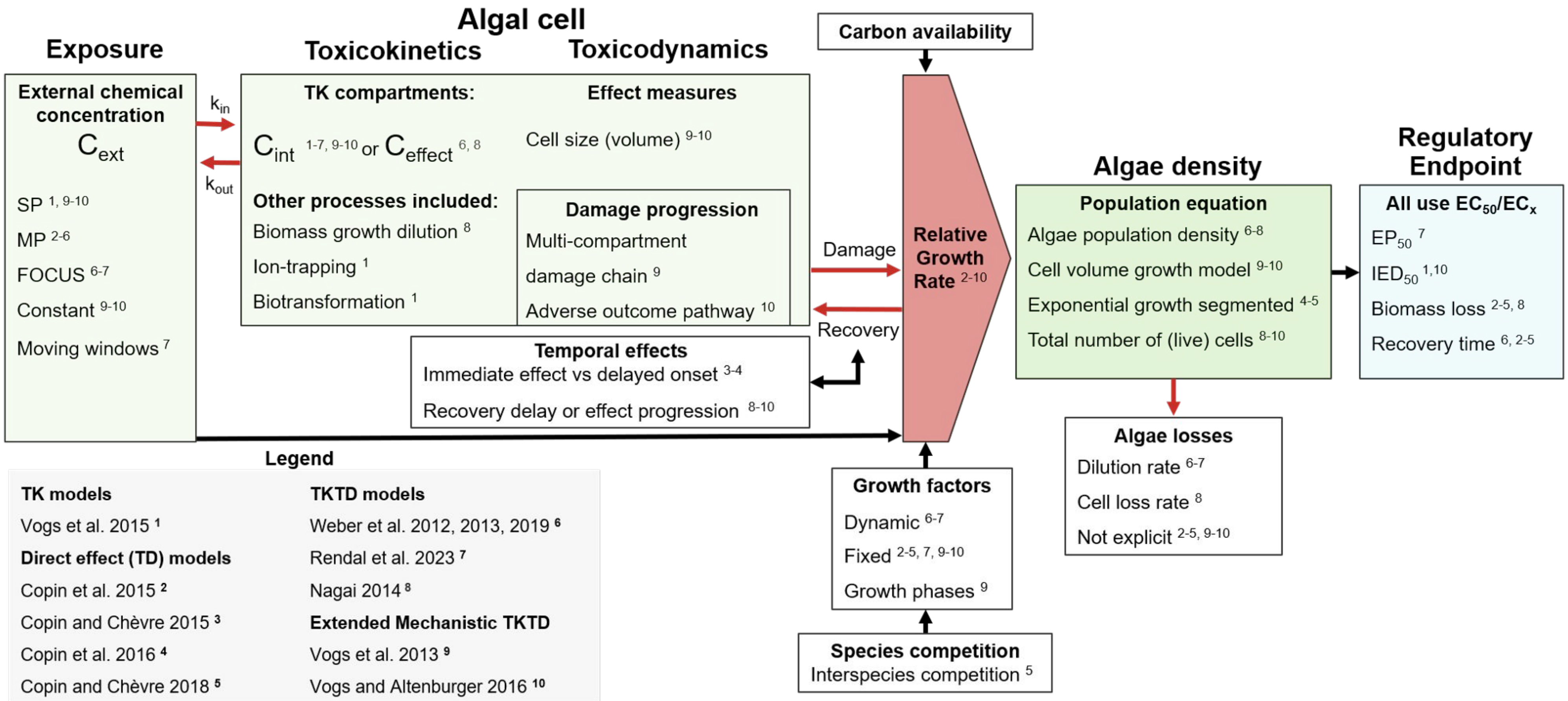

Figure S2. Comparative overview of the parameters (state-variables, boxes) and processes (rate constants, red arrows) included in the 12 reviewed algae growth models. The framework has been organized into five modules: exposure, algal cell (toxicokinetic and toxicodynamic processes), relative growth rate, algae density, and regulatory endpoints. The grayscale shapes are associated with the studies reviewed in the papers under the four main model type groupings: TK models, direct effect (TD) models, TKTD models, and extended mechanistic TKTD. In the exposure module, single pulse (SP) and multiple pulses (MP), as described in Fig. 2, are included. These exposure descriptions describe what was performed in the papers, but not what the models are limited to. Between the exposure module and toxicokinetics, the uptake (k_{in}) and elimination (k_{out}) rate constants were determined in the models using effective concentrations (C_{effect}) as opposed to the assumption that the external chemical concentration (C_{ext}) was equivalent to the internal concentration (C_{int}). The C_{int} / C_{effect} then affects algae growth rates (RGR), which can be described at different levels of detail as shown in the Toxicodynamic module. Other factors affecting algae growth rates are given in the module “Growth factors” and in Table 2. Jointly, they feed into algae density as a function of time (Module: relative growth rate). From the modelled algae densities at specific points in time, a range of regulatory endpoints can be derived. Effect concentrations of x% EC_x are given in all studies, but also other endpoints, such as the multiplication factor required to cause 50% growth inhibition (EP_{50}) of a specific variable exposure profile within a selected time-window, are given in addition to the internal effect concentration of a chemical in the whole organism (IED_{50}).
